# Robenacoxib versus meloxicam for the control of peri-operative pain and inflammation associated with orthopaedic surgery in cats: a randomised clinical trial

**DOI:** 10.1186/s12917-015-0391-z

**Published:** 2015-03-26

**Authors:** Cindy Speranza, Vincent Schmid, Jerome M Giraudel, Wolfgang Seewald, Jonathan N King

**Affiliations:** Novartis Santé Animale S.A.S, Clinical Development, F-92506 Rueil Malmaison Cedex, France; Novartis Centre de Recherche Santé Animale SA, CH-1566 Saint-Aubin, Switzerland; Novartis Animal Health Inc, Project Management, CH-4058 Basel, Switzerland; Novartis Animal Health Inc, Clinical Development, CH-4058 Basel, Switzerland

**Keywords:** Cat, Coxib, Non-steroidal anti-inflammatory drug, Pain, Peri-operative, Robenacoxib

## Abstract

**Background:**

Non-steroidal anti-inflammatory drugs (NSAIDs) are widely used in veterinary medicine. Robenacoxib is a NSAID with high selectivity for the cyclo-oxygenase-2 enzyme. In this study, the efficacy and safety of robenacoxib were evaluated in a prospective, randomised, active- and placebo-controlled masked clinical trial in 147 cats undergoing orthopaedic surgery. Cats were randomised into two treatment groups: Group 1, robenacoxib (2 mg/kg) administered via subcutaneous (s.c.) injection before surgery, followed by robenacoxib tablets (1–2.4 mg/kg) administered post-operatively for approximately 9 days (n = 101) and Group 2, meloxicam (0.3 mg/kg) administered s.c. before surgery, followed by placebo tablets administered post-operatively for approximately 9 days (n = 46). Cats were assessed using numerical rating scales (NRSs) by clinicians before surgery and at 3, 8, 22 and 28 hours after surgery and at the final visit (VF on approximately Day 10), and daily by their owners from Day 1 to the VF.

**Results:**

The primary end point was the global investigator score which was the sum of clinician NRSs for posture, behaviour and pain on palpation/manipulation. The efficacy of the single robenacoxib injection, assessed during 3 to 22 hours, was statistically non-inferior to meloxicam, with a relative efficacy of 1.029 (95% confidence interval, 0.847–1.231). No significant differences were detected during the follow-up treatment with robenacoxib tablets for approximately 9 days compared with placebo via clinician assessments at 28 hours and the VF, or in owner assessments on Days 1–VF. There were no significant differences in frequencies of reported adverse events, clinical observations and haematology or clinical chemistry variables between the groups.

**Conclusions:**

Single s.c. injection of robenacoxib before surgery had non-inferior efficacy compared with meloxicam in controlling post-operative pain and inflammation in cats undergoing orthopaedic surgery. Follow-up treatment with oral robenacoxib tablets for approximately 9 days was well tolerated, but there were no differences in the efficacy scores after Day 1 compared with the group receiving meloxicam s.c. followed by placebo control.

## Background

Non-steroidal anti-inflammatory drugs (NSAIDs) are commonly used to manage pain, inflammation and fever [1]. Despite the recognised need for effective drugs of this class in cats, there are relatively few preclinical and clinical studies on NSAIDs in this species and fewer approved NSAIDs in cats compared with dogs [1]. One reason for the limited availability of feline NSAIDs is the low safety indices of many NSAIDs in cats [1].

Robenacoxib is a NSAID of the Coxib class which is registered for use in cats and dogs. Owing to its availability as injection and tablet formulations with proven efficacy and good safety, robenacoxib has an interesting profile for use in cats. Robenacoxib is rapidly absorbed after subcutaneous (s.c.) injection and oral administration in cats, with mean time to maximal concentration (T_max_) values of 1 and 0.5 hours, respectively [2]. Robenacoxib demonstrated analgesic, anti-inflammatory and anti-pyretic activity in cats in a kaolin-induced paw inflammation model [3]. In clinical trials, non-inferior efficacy of robenacoxib compared with ketoprofen was observed in acute musculoskeletal disorders [4,5] and superior efficacy against pain was reported versus meloxicam in cats undergoing surgery [6]. Superiority of robenacoxib tablets versus placebo was also demonstrated in cats undergoing surgery [7]. Robenacoxib has a high safety index in healthy cats: oral dosages up to 20 mg/kg/day for 42 days were well tolerated compared with the recommended dosage of 1–2.4 mg/kg [8]. The good safety profile of robenacoxib may be attributed to its combination of enzyme specificity (for cyclo-oxygenase-2 (COX-2)), short half-life (~1.5 hour) in blood and selective tissue distribution [8,9].

The aim of the present investigation was to evaluate the field efficacy and safety of robenacoxib in controlling peri-operative pain and inflammation associated with orthopaedic surgery in cats. Robenacoxib (s.c. followed by tablets) was compared with meloxicam (s.c. followed by placebo tablets). Meloxicam is a preferential inhibitor of COX-2, with a long terminal plasma half-life in cats [1,9,10].

## Methods

### Study design

The study was a multi-centre, prospective, randomised, masked, parallel-group design clinical trial comparing two treatment groups. Group 1 received robenacoxib by s.c. injection pre-operatively, followed by robenacoxib tablets administered post-operatively. Group 2 received pre-operative meloxicam by s.c. injection, followed by post-operative placebo tablets. Meloxicam was selected as the active control as meloxicam 5 mg/mL solution for injection is registered for single use at a dose of 0.3 mg/kg, administered before surgery, for the reduction of post-operative pain after ovariohysterectomy and minor orthopaedic surgery in the European Union (EU), and for the control of post-operative pain and inflammation associated with orthopaedic surgery, ovariohysterectomy and castration in the United States (US). At the time the study was initiated, meloxicam was not registered for post-operative use in cats in the EU (or the US). Therefore robenacoxib (s.c. injection followed by tablets) was compared to single pre-operative meloxicam by s.c. injection followed by administration of placebo tablets. The administration of placebo tablets to Group 2 was considered acceptable on welfare grounds because of the absence of registered products at the time of the study and the fact that meloxicam has a long duration of action in cats [10]. Clinicians were instructed to administer additional analgesia of their choice as rescue therapy at any time, if judged to be needed.

The study was authorised by the French (Agence Nationale du Médicaments Vétérinaire, authorization # EC/05/043) and United Kingdom (UK) (Veterinary Medicines Directorate, Animal Test Certificate Vm12501/0038) regulatory authorities and internal Novartis reviews based on scientific, ethical and animal welfare guidelines.

The study was also conducted in compliance with: (a) the EMEA/CVMP/237/01 document, Guideline for the Conduct of Efficacy Studies for Non-Steroidal Anti-Inflammatory Drugs; (b) the document, Procedures and Principles of Good Clinical Practice as detailed in the Veterinary International Conference on Harmonization Guideline on Good Clinical Practices (GL9)—CVMP/VICH/595/98; and (c) the Guideline on Statistical Principles for Veterinary Clinical Trials (EMEA/CVMP/816/00). All owners gave written informed consent to include their cats in the study.

### Animals

The inclusion criteria for cats were age ≥6 weeks, body weight 2.5–12 kg, either sex, any breed, presenting for major orthopaedic surgery under general anaesthesia. The exclusion criteria for cats were pregnant or lactating or females intended for breeding; with severe concomitant disorders (e.g., gastrointestinal disease, kidney or liver insufficiency); treated before study commencement with local or systemic NSAIDs or opioids (within 24 hours, apart from butorphanol in the UK as part of the pre-anaesthetic regimen), corticosteroids (short-acting systemic or locally acting within 30 days, long-acting within 60 days); and undergoing repeated surgeries within a few days.

### Randomisation and blinding procedures

Animals were randomly assigned to the two treatment groups in a 2:1 robenacoxib:meloxicam ratio from computer-generated randomisation lists. The 2:1 ratio was used to obtain data in more cats receiving robenacoxib and was predicted to cause only a modest (approximately 10%) reduction in statistical efficiency compared to a 1:1 ratio. Case allocation was stratified according to the investigation centre and the anticipated duration of surgery (<45 minutes, >45 minutes). Investigators were blinded to the block length, which was six. The robenacoxib and meloxicam solutions for injection have a different appearance. Masking was therefore maintained by the ‘blinding by function’ technique: a clinician was responsible for clinical assessments, whereas a separate dispenser was responsible for treatment prescription, administration and compliance control. For the oral treatment, both placebo and robenacoxib tablets had the same appearance and packaging, therefore owners were blinded to the treatment.

### Drugs and administration procedures

Investigational drugs were a parenteral formulation of robenacoxib^a^, a tablet formulation of robenacoxib^b^, a parenteral formulation of meloxicam^c^ and placebo tablets^d^. Target and actual dosages are listed in Table 1. The s.c. injections of robenacoxib and meloxicam were given between the shoulder blades, shortly before the induction of anaesthesia. Owners were instructed to administer tablets either with no food or with only a small quantity of food. Cats received once daily one (body weight, 2.5–6.0 kg) or two (body weight, >6.0-12.0 kg) tablets containing robenacoxib or placebo for 9 days (range, 7–11 days). There was no mandatory anaesthetic regimen, but times of anaesthetic induction, duration of anaesthesia and recovery from anaesthesia were recorded.

**Table 1:** **Treatment groups**

**Groups**	**Number of cats**	**s.c. dosage (mean ± SD) in mg/kg**		Daily oral dosage (mean ± SD) in mg/kg*	
		**Target**	**Actual**	**Target**	**Actual**
Robenacoxib (s.c. + oral)	101	2.0	2.0 ± 0.09	1.0 – 2.4	1.58 **±** 0.41
Meloxicam (s.c. + placebo oral)	46	0.3	0.3 ± 0.005	-	-

s.c.: subcutaneous.

*Robenacoxib and placebo tablets were administered once daily for 9 (range, 7–11) days.

All concomitant treatments were recorded. The use of fluid therapy and antibiotics was permitted. Drugs likely to affect efficacy assessments, including all classes of analgesics (except those incorporated in the pre-anaesthetic regimen) including other NSAIDs or corticosteroids were forbidden, except as rescue therapy.

### Study schedule

The study schedule is outlined in Table 2.

**Table 2:** **Dosing and monitoring schedule**

	**Day 0**					**Day 1**			**Day 2 to 9**	**Day 10**
Time (T)	Initial visit	T0*	T1^a^	T1 + 3 hours (±0.5 hours)	T1 + 8 hours (±1 hours)	T1 + 22 hours (±2 hours)	T2^b^	T2 + 4 hours (±2 hours)		Final visit (±2 days)
Description	V1			V2	V3	V4		V5		VF
Location	Clinic	Clinic	Clinic	Clinic	Clinic	Clinic	Clinic	Clinic	Home	Clinic
Activity		Administer s.c. injection Anaesthesia induction	Extubation				First oral dose		Once-daily oral doses^c^	

s.c.: subcutaneous; V: visit; VF: final visit.

^*^The s.c. first drug administration was close to the time of anaesthetic induction (T0).

^a^Time of extubation if the cat was intubated during anaesthesia or return of palpebral reflex if not intubated (T1).

^b^The first tablet was administered by the dispenser approximately 24 hours after the s.c. administration (T2).

^c^Tablets were administered from Day 2 to the day preceding the VF by the owner. The total duration of oral dosing was 9 days (range, 7–11 days).

### Efficacy evaluation criteria

Cats were assessed by a clinician while the cat was hospitalised pre-surgery (visit (V)1) and post-surgery (V2 to the final visit (VF)) (Table 2) for pain, posture and behaviour (Table 3). In addition, each owner assessed the cat’s activity, appetite, behaviour and the interaction with the owner and/or other people in their home environment daily from Day 1 to VF (Table 4).

**Table 3:** Summary of numerical rating scales (lower and upper descriptors only) used by the investigator (clinician)*

**Variable**	**Lower descriptor (0)**	**Upper descriptor (3)**
Posture of cat in recovery cage^a^	Comfortable, relaxed	Rigid or tense
Behaviour of cat in recovery cage^a^	Alert, positive interaction with clinician when cage is opened	Depressed, aggressive or non-responsive to stimulation
Pain elicited on manipulation of wound or area of operation^b^	No reaction to even strong manipulation	Extreme reaction to any manipulation
Overall pain control^c^	Excellent	Poor

*Each variable was assessed on a 4-point scale (0 to 3).

^a^Assessments at V1 to V5.

^b^Assessments at V1 to V5 and VF (final visit).

^c^Assessments at V2 to V5 and VF.

All four end points were secondary endpoints in the statistical analysis. The posture, behaviour and pain on manipulation scores were summed for each animal to provide the primary end point, the global investigator score.

**Table 4:** Summary of the numerical rating scales (lower and upper descriptors only) used by the owner*

**Variable**	**Lower descriptor (0)**	**Upper descriptor (3)**
Level of activity compared with normal^a^	Normal	Severely impaired/depressed
Animal behaviour^a^	Normal, alert, content	Depressed
Appetite^a^	Good (normal or improved)	Not eating
Interactions with owner, other humans, other animals (sociability)^a^	Good (normal or improved)	None, unresponsive
Palatability of tablets^b^	Excellent, voluntary intake from owner’s hand or food/bowl	Reluctance to accept all dosing procedures

*Each variable was assessed on a 4-point scale (0 to 3) except palatability (0 to 4).

^a^Assessments from Day 1 to VF (final visit). The sum of the 4 scores was used to derive a multi-dimensional rating assessment, the global owner score. The assessment form at VF was completed by the investigator and owner together.

^b^Assessment each day from Day 1 to day preceding VF; on Day 1 by the dispenser, on all other days by the owner.

The primary efficacy end point was the global investigator score (described as a multi-dimensional rating scale), which was the non-weighted sum of the posture, behaviour and pain on palpation/manipulation numerical rating scales (NRS) assessed by the clinician (Table 3). All other efficacy assessments were secondary efficacy end points.

### Plasma cortisol concentration

Blood samples were collected at time (T)0, T1, V5 and VF (Table 2). Plasma cortisol concentrations were determined using a commercial radio-immunoassay kit (IMMULITE® 2000 Cortisol kit, Siemens Healthcare Diagnostics, NY, US) by personnel at the National Veterinary School of Toulouse, France. Within-day and between-day precisions were < 14%, and the accuracy ranged from 93% to 109%. The limit of quantitation of the assay was 10 ng/mL.

### Tolerability

The clinician examined each cat and checked for vomiting and diarrhoea on the floor of the recovery cage at V2, V3, V4 and V5 and, in addition, examined the cat at VF. Owners were requested to report all adverse events occurring from Day 1 to VF.

### Pain and inflammation at the injection site

Local tolerance at injection sites was assessed by the dispenser using 4-point NRSs (range, 0–3) for which the lower and upper descriptors were: (a) pain (0 = none, 3 = severe) at T0; (b) inflammation (0 = none, 3 = severe) at T2; and (c) pain on palpation (0 = no reaction, 3 = extreme reaction) at T2. These assessments were not blinded.

### Clinical chemistry and haematology

Blood samples for plasma clinical chemistry (into heparin) and haematology (into EDTA) were taken before anaesthetic induction and at V5 and VF. Variables included activities of alanine aminotransferase (ALT), aspartate aminotransferase (AST), alkaline phosphatase (AP) and creatine kinase (CK), concentrations of albumin, creatinine, potassium, sodium, total protein and urea, red blood cell count, white blood cell count, differential white cell count, platelet count, haematocrit and haemoglobin concentration.

### Palatability

The palatability of the tablets was assessed by the dispenser at V5 on Day 1 and by the owners from Day 2 to the day preceding the VF using a 5-point NRS (Table 4).

### Statistics

Data are presented as mean (SD). All analyses were based on the ‘intention-to-treat’ data set, that is all randomised animals that received at least the s.c. injection and from which at least one measurement post-treatment was obtained. Statistical tests were performed using SAS® Software version 8.2 (SAS Online Doc, Version 8, Cary NC, US; SAS Institute Inc, 1999).

Groups were compared at baseline using the Mann–Whitney *U* test. Efficacy, cortisol, clinical chemistry and haematology variables were analysed using repeated measures analysis of covariance (RMANCOVA). Variables were transformed (log or reciprocal), if appropriate, to give the best estimate of a normal distribution which was assessed using the Shapiro-Wilk test.

The RMANCOVA model contained the model parameters: treatment group, baseline value, time, weight, age, investigator/owner, duration of intubation and type of surgery. Model parameters (except treatment group) that gave a *P* value of >0.05 were successively deleted from the model. Non-inferiority for efficacy variables was concluded when the lower limit of the 95% confidence interval (CI) for the ratio robenacoxib/meloxicam was higher than 1–δ, with δ defined as 0.25 [11].

As many variables deviated significantly from a normal distribution in the RMANCOVA analysis, groups were in addition compared with the non-parametric Mann–Whitney test. Change from baseline was assessed using the Wilcoxon paired-samples test. The occurrence of adverse events in the two treatment groups was compared using the Fisher exact probability test. All statistical tests were two-sided on a 5% level of significance (α = 0.05).

## Results

### Baseline and demographic variables

A total of 147 cats were recruited at 21 investigator sites from different practices in France and the UK between December 2005 and August 2007. No cases were removed from the analysis, leaving 147 cats in the analysed data set. The number of cases per centre ranged from 1 to 22 (robenacoxib 0 to 14, meloxicam plus placebo 0 to 8, *P* = 0.91).

There were no significant differences between the two groups in baseline, surgery or anaesthetic variables (Table 5). The duration of surgery was 60 minutes or less in 69% of cases. The majority (51%) of cats underwent fracture repair.

**Table 5:** **Baseline, surgery and anaesthetic variables**

**Variable**	**Robenacoxib (s.c. + oral)**	**Meloxicam s.c. + placebo oral**	**Total**	***P*** **value**
**Total**	101	46	147	
**Age**	3.8 (4.1)	2.9 (3.1)	3.5 (3.8)	0.075
**Body weight**	4.0 (1.0)	3.8 (1.0)	3.9 (1.0)	0.26
**Sex and neutered status**				
Male not neutered	22 (22%)	16 (35%)	38 (26%)	0.19
Female not neutered	12 (12%)	8 (17%)	20 (14%)	
Male neutered	38 (38%)	15 (33%)	53 (36%)	
Female neutered	29 (29%)	7 (15%)	36 (24%)	
**Breed**				
Burmese	1 (1%)	0 (0%)	1 (1%)	0.37
Crossbred	3 (3%)	0 (0%)	3 (2%)	
Domestic long hair	5 (5%)	0 (0%)	5 (3%)	
Domestic short hair	33 (33%)	15 (33%)	48 (33%)	
European	57 (56%)	29 (63%)	86 (59%)	
Persian	1 (1%)	0 (0%)	1 (1%)	
Siamese	1 (1%)	2 (4%)	3 (2%)	
**Baseline clinician scores**				
Global investigator score	3.1 (1.9)	3.5 (2.3)	3.2 (2.0)	0.50
Pain at palpation/manipulation	1.5 (0.8)	1.6 (0.8)	1.5 (0.8)	0.44
**Expected duration of surgery (hours)**				
<60 min	71 (70%)	30 (65%)	101 (69%)	0.57
>60 min	30 (30%)	16 (35%)	46 (31%)	
**Effective duration of surgery (hours)**	53.0 (38.8)	57.9 (39.2)	54.6 (38.8)	0.41
**Effective duration of intubation**	80.4 (48.5)	79.5 (50.0)	80.2 (48.8)	0.82
**Type of surgery**				
Fracture, internal repair	49 (49%)	26 (57%)	75 (51%)	0.38
Fracture, no internal repair	1 (1%)	0 (0%)	1 (1%)	1.0
Hip surgery	10 (10%)	4 (9%)	14 (10%)	1.0
Amputation, limb	6 (6%)	0 (0%)	6 (4%)	0.18
Amputation, tail or phalanx	6 (6%)	4 (9%)	10 (7%)	0.73
Joint surgery	13 (13%)	6 (13%)	19 (13%)	1.0
Removal of osteosynthesis material	13 (13%)	3 (7%)	16 (11%)	0.28
Miscellaneous	2 (2%)	2 (4%)	4 (3%)	0.59
Combinations	1 (1%)	1 (2%)	2 (1%)	1.0
**Substances used in anaesthesia**				
Acepromazine	22 (22%)	12 (26%)	34 (23%)	0.67
Butorphanol	33 (33%)	15 (33%)	48 (33%)	1.0
Diazepam	6 (6%)	4 (9%)	10 (7%)	0.73
Ketamine	57 (56%)	28 (61%)	85 (58%)	0.72
Medetomidine	54 (53%)	17 (37%)	71 (48%)	0.076
Medetomidine/acepromazine	0 (0%)	1 (2%)	1 (1%)	0.31
Propofol	33 (33%)	12 (26%)	45 (31%)	0.450
Thiopental	5 (5%)	3 (7%)	8 (5%)	1.0
Tiletamine/zolazepam	7 (7%)	2 (4%)	9 (6%)	0.72
Xylazine	18 (18%)	11 (24%)	29 (20%)	0.50

s.c.: subcutaneous.

Data are mean (SD) or number of cats (%).

### Efficacy

There were no significant differences between groups in the number of cats which received additional analgesics as rescue therapy (robenacoxib n = 2 (2%), meloxicam n = 2 (4%), *P* = 0.56) or which were withdrawn because of judged lack of efficacy (robenacoxib n = 4 (4%), meloxicam n = 0 (0%), *P* = 0.23). The rescue therapy was administered by injection in all cases: 0.09 mg buprenorphine and 1 mg methadone respectively in the two cats in the robenacoxib group and 0.05 and 0.5 mg morphine respectively in the two cases in the meloxicam group.

### Efficacy in the first 24 hours after surgery

**Figure 1:**
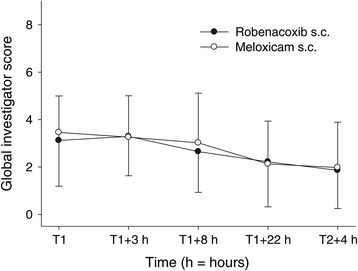
**Mean (±SD) global investigator scores at assessment times V1 to V5.** For an explanation of the global investigator scores (0 = best possible, 9 = worst possible) see Table 3.

The efficacy of the injectable products was assessed by comparing clinician assessments in the first 24 hours (V2 to V4). Scores were numerically but not statistically significantly lower (better) with robenacoxib for the primary end point and three of the five secondary end points (Table 6). The global investigator scores were low at all time points in both the groups and decreased with time after T1 + 3 hours (Figure 1).

**Table 6:** **Summary statistics for the clinician efficacy scores in the first 24 hours after surgery**

**Response**	**Visits**	**Robenacoxib (s.c. + oral)**	**Meloxicam s.c. + oral placebo**	***P*** **value (Mann–Whitney test)**	**Quotient robenacoxib:meloxicam (RMANCOVA)**		***P*** **value (RMANCOVA)**	**Transformation for RMANCOVA analysis**	
		**Mean (SD)**	**Mean (SD)**		**Mean**	**95% CI**		**Transformation**	***P*** **value for normality (Shapiro-Wilks test)**
Global investigator score (primary end point)	V2-V4	2.72 (1.42)	2.80 (1.62)	0.76	1.029	**0.847–1.231**	0.76	0 (log)	0.20
Posture	V2-V4	0.68 (0.53)	0.79 (0.61)	0.38	1.144	**0.821–1.538**	0.39	−1 (reciprocal)	**<0.0001**
Behaviour	V2-V4	0.81 (0.56)	0.88 (0.64)	0.91	1.073	**0.827–1.360**	0.58	−0.5 (reciprocal of square root)	**0.024**
Pain on palpation/manipulation	V2-V4	1.22 (0.69)	1.14 (0.69)	0.23	0.979	**0.799–1.184**	0.82	0.5 (square root)	**0.0024**
Overall pain control	V2-V4	1.17 (0.76)	1.15 (0.67)	0.89	1.120	**0.880–1.408**	0.34	0.5 (square root)	**0.011**
Sedation	V2-V4	0.44 (0.44)	0.40 (0.42)	0.76	0.945	0.396–1.755	0.85	−0.5 (reciprocal of square root)	**0.024**

CI: confidence interval; s.c.: subcutaneous.

Values are mean (SD) for each group and quotient values with 95% CIs for the ratio robenacoxib/meloxicam. Data are from the assessments at V2, V3 and V4. Non-inferiority of robenacoxib versus meloxicam was concluded if the lower limit of the 95% CI was >0.75 (shown in bold). P values <0.05 are also shown in bold.

The global investigator score (the primary end point) ranged from 0 to 9. All other end points (secondary end points) ranged from 0 to 3.

Non-inferiority analysis was conducted using RMANCOVA. However, in spite of the transformations, only the primary efficacy end point, the global investigator score fulfilled normal distribution assumptions (*P* = 0.20). Non-inferiority of robenacoxib versus meloxicam was demonstrated for the global investigator score, with the relative efficacy of robenacoxib versus meloxicam being 1.029 (95% CI, 0.847–1.231). The effect of covariates was significant for baseline, investigator and time (*P* <0.001), but was non-significant for age, body weight, country, duration of intubation, treatment, treatment × time interaction and type of surgery.

All secondary end points deviated from a normal distribution (*P* <0.05) in the RMANCOVA even after transformations (Table 6). For the primary and secondary clinician end points, there were no significant differences between groups in the non-parametric Mann–Whitney comparisons (Table 6).

### Efficacy from Day 1 to the VF after surgery

The efficacy of the robenacoxib tablets compared with the placebo tablets was assessed from the clinicians’ assessments at T2 + 4 hours (28 hours) and VF (Table 7) plus the owners’ assessments on Day 1 to VF (Table 8). In the RMANCOVA analyses, only the global investigator score was normally distributed after transformation (*P* = 0.53). Clinician scores were numerically lower with robenacoxib than meloxicam + placebo for the five secondary endpoints but not the primary endpoint, however differences were not significant using the non-parametric Mann–Whitney test (Table 7).

**Table 7:** **Summary statistics for the clinician efficacy scores assessed more than 24 hours after surgery**

**Response**	**Visits**	**Robenacoxib (s.c. + oral)**	**Meloxicam s.c. + oral placebo**	***P*** **value (Mann–Whitney test)**	**Quotient robenacoxib:meloxicam (RMANCOVA)**		***P*** **value (RMANCOVA)**	**Transformation for RMANCOVA analysis**	
		**Mean (SD)**	**Mean (SD)**		**Mean**	**95% CI**		**Exponential**	***P*** **value for normality (Shapiro-Wilks test)**
Global investigator score (primary end point)	V5	1.86 (1.61)	1.98 (1.91)	0.93	0.968	0.682–1.316	0.84	0 (log)	0.53
Posture	V5	0.70 (0.68)	0.78 (0.74)	0.72	1.007	0.527–1.693	0.98	−1 (reciprocal)	**<0.0001**
Behaviour	V5	0.42 (0.59)	0.54 (0.84)	1.0	1.064	0.565–1.786	0.82	−0.5 (reciprocal of square root)	**<0.0001**
Pain on palpation/manipulation	V5 & VF	0.44 (0.62)	0.52 (0.84)	0.65	1.032	**0.768–1.343**	0.82	0.5 (square root)	**<0.0001**
Overall pain control	V5 & VF	0.76 (0.62)	0.76 (0.71)	0.49	1.143	**0.807–1.567**	0.42	0.5 (square root)	**<0.0001**
Inflammation intensity	VF	0.15 (0.04)	0.25 (0.06)	0.16	1.642	**0.752–3.695**	0.18	0 (log)	**<0.0001**

CI: confidence interval; s.c.: subcutaneous; VF: final visit.

Values are mean (SD) for each group and quotient values with 95% CI for the ratio robenacoxib/meloxicam. Data are from assessments at V5 and VF.

Non-inferiority of robenacoxib versus meloxicam was concluded if the lower limit of the 95% CI was >0.75 (shown in bold). P values <0.05 are also shown in bold.

The global investigator score (the primary end point) ranged from 0 to 9. All other end points (secondary end points) ranged from 0 to 3.

**Table 8:** **Summary statistics for the owner efficacy scores from Day 1 to VF**

**Response**	**Visits**	**Robenacoxib (s.c. + oral)**	**Meloxicam s.c. + oral placebo**	***P*** **value (Mann–Whitney test)**	**Quotient robenacoxib:meloxicam (RMANCOVA)**		***P*** **value (RMANCOVA)**	**Transformation for RMANCOVA analysis**	
		**Mean (SD)**	**Mean (SD)**		**Mean**	**95% CI**		**Exponential**	***P*** **value for normality (Shapiro-Wilks test)**
Level of activity	D1-VF	0.62 (0.53)	0.64 (0.61)	0.96	0.972	0.658–1.373	0.87	0 (log)	**<0.0001**
Behaviour	D1-VF	0.29 (0.38)	0.31 (0.34)	0.57	1.285	0.731–2.240	0.34	−1 (reciprocal)	**<0.0001**
Appetite	D1-VF	0.38 (0.48)	0.37 (0.49)	0.69	1.006	0.551–1.672	0.98	−1 (reciprocal)	**<0.0001**
Interaction	D1-VF	0.23 (0.34)	0.26 (0.33)	0.70	1.306	0.696–2.452	0.36	−1 (reciprocal)	**<0.0001**
Global owner score	D1-VF	1.52 (1.42)	1.58 (1.45)	0.87	1.050	0.719–1.482	0.78	0 (log)	**<0.0001**

CI: confidence interval; VF: final visit.

Values are mean (SD) for each group and quotient values with 95% CI for the ratio robenacoxib/meloxicam. All assessments made by the owners are secondary end points. Assessments were made daily from the day after surgery (Day 1) to VF.

Non-inferiority of robenacoxib versus meloxicam was concluded if the lower limit of the 95% CI was >0.75 (shown in bold). P values <0.05 are also shown in bold.

The level of activity, behaviour, appetite and interaction scores ranged from 0 to 3. The global owner score ranged from 0 to 12.

**Figure 2:**
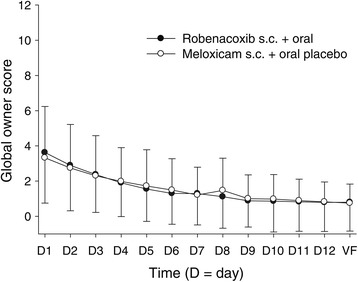
**Mean (±SD) global owner scores from Day 1 to the VF.** For an explanation of the global owner score (0 = best possible, 12 = worst possible) see Table 4.

The scores were low in both the groups for all variables for the owner’s end points. In the RMANCOVA analyses, all variables deviated markedly from a normal distribution (*P* <0.001). Scores were numerically lower with robenacoxib than meloxicam + placebo for four of the five owners’ variables, but differences were not significant with the non-parametric Mann–Whitney test for any variable (Table 8). The global owner score results are shown in Figure 2.

### Plasma cortisol concentration

Cortisol concentrations (nmol/L; mean ± SD) for Group 1 (n = 60) and Group 2 (n = 29), respectively, were 106.1 ± 74.6 and 92.9 ± 61.5 at T0; 106.3 ± 79.8 and 109.8 ± 68.0 at T1; 90.9 ± 87.6 and 87.8 ± 61.8 at V5; and 87.2 ± 73.6 and 90.6 ± 54.9 at VF. Thus, inter-animal variation in concentration was high at all times in both the groups. The only significant change from baseline was lower concentrations at V5 in the robenacoxib group (*P* = 0.024). Differences between groups were not statistically significant. Non-inferiority was demonstrated in the RMANCOVA analysis, the relative efficacy (95% CI), calculated from the reciprocal of cortisol concentrations, of robenacoxib versus meloxicam + placebo was 1.148 (0.896–1.445).

### Tolerability

The numbers of cats with reported adverse events, which may or may not have been related to the robenacoxib, meloxicam and placebo treatments, were 30/101 (30%) for the robenacoxib group and 12/46 (26%) for the meloxicam + placebo group (*P* = 0.70, Table 9).

**Table 9:** **Reported adverse events**

**Response**	**Robenacoxib (s.c. + oral)**	**Meloxicam s.c. + oral placebo**	***P*** **value (Fisher exact test)**
Abdominal cavity hernia	0/101 (0%)	1/46 (2%)	0.31
Anorexia	1/101 (1%)	0/46 (0%)	1.0
Blood in faeces	1/101 (1%)	1/46 (2%)	1.0
Diarrhoea	7/101 (7%)	3/46 (7%)	1.0
Disorientation	1/101 (1%)	0/46 (0%)	1.0
Emesis	13/101 (13%)	5/46 (11%)	0.79
Eye redness	1/101 (1%)	0/46 (0%)	1.0
Loose stool	9/101 (9%)	4/46 (9%)	1.0
Muscle tremor	1/101 (1%)	0/46 (0%)	1.0
Pain not otherwise specified	0/101 (0%)	1/46 (2%)	0.31
Polydipsia	0/101 (0%)	1/46 (2%)	0.31
Prostration	1/101 (1%)	0/46 (0%)	1.0
Prolonged recovery	1/101 (1%)	0/46 (0%)	1.0
Sneezing	1/101 (1%)	0/46 (0%)	1.0
Spasm	0/101 (0%)	1/46 (2%)	0.31
Vocalisation	0/101 (0%)	1/46 (2%)	0.31
Uncoded sign	2/101 (2%)	1/46 (2%)	1.0
Any adverse event	30/101 (30%)	12/46 (26%)	0.70

s.c.: subcutaneous.

Data are number of cats with reported adverse event/total number of cats (%) in the whole study.

A higher than 2% incidence of reported adverse events was described only for diarrhoea, loose stool and emesis. The reported frequency in the robenacoxib and meloxicam + placebo groups, respectively, was 7% and 7% for diarrhoea (*P* = 1.0), 9% and 9% for loose stool (*P* = 1.0), and 13% and 11% for emesis (*P* = 0.79).

### Pain and inflammation at the injection site

The dispenser assessed (unblinded) the pain during the s.c. injection. The mean (SD) scores were 0.14 (0.43) with robenacoxib and 0.16 (0.48) with meloxicam. Differences were not significant (*P* = 0.87). At T2 (approximately 24 hours after the s.c. injection), the dispenser assessed inflammation and pain at palpation at the original site of injection. The mean (SD) scores for robenacoxib and meloxicam were, respectively, 0.05 (0.22) and 0.05 (0.21) for inflammation (*P* = 1.0), and 0.03 (0.17) and 0.05 (0.21) for pain on palpation (*P* = 1.0) (data not shown).

### Clinical chemistry

At V5 (28 hours post-surgery), the following changes from baseline were significant: in both the groups, AST, CK and potassium were increased, whereas AP and albumin were decreased; in the robenacoxib group, creatinine was decreased; and in the meloxicam group, urea was increased and total protein was decreased. At VF in both the groups, ALT, AST and CK were significantly decreased compared with baseline and AP, creatinine, sodium and urea were significantly increased. Differences between groups were significant for creatinine and urea with higher values in the meloxicam + placebo group (data not shown).

### Haematology

At V5 (28 hours post-surgery), red and white cell counts, haemoglobin and haematocrit were significantly increased compared with baseline in the robenacoxib group. At VF, red cell counts, haemoglobin and haematocrit were significantly increased compared with baseline in the robenacoxib group. The only significant change from baseline in the meloxicam + placebo group was a decrease in white cell counts at the VF. The only significant difference between the two treatment groups was lower white cell counts in the meloxicam group (data not shown).

### Palatability

The palatability of the tablets containing robenacoxib was compared with placebo by the dispenser at V5 on Day 1 and by the owners daily from Day 2 to the VF. Differences between groups were not significant. The mean (SD) scores in the robenacoxib and placebo groups were, respectively, 1.46 (1.03) and 1.28 (0.88) for the dispenser (*P* = 0.38) and 1.15 (1.09) and 1.16 (0.88) for the owner (*P* = 0.54).

## Discussion

The principal finding of this study is that single s.c. injection of robenacoxib before surgery was well tolerated and had statistically non-inferior efficacy in comparison with meloxicam for the management of pain and inflammation associated with orthopaedic surgery in cats. Both drugs provided good efficacy, as evidenced from low scores for pain and inflammation post-surgery and low frequency of rescue therapy.

The primary end point, the global investigator score, was the unweighted sum of three secondary end points (behaviour, posture and pain on palpation). This end point fulfilled normal distribution assumptions after log transformation, permitting non-inferiority of the robenacoxib injection versus meloxicam to be tested (and demonstrated) using the powerful parametric RMANCOVA analysis. The relative efficacy (95% CI) of robenacoxib versus meloxicam was 1.029 (0.847-1.231), showing numerical but not significantly superior efficacy of robenacoxib. All five secondary investigator end points showed significant deviation from a normal distribution even after transformation, therefore statistical comparison between groups for these end points had to be based on lack of significant differences using the non-parametric Mann–Whitney test.

Meloxicam injection was selected as the positive control as it is registered and extensively used, and its efficacy is documented in the literature in cats. The superiority of meloxicam by injection at the 0.3 mg/kg dose versus placebo was demonstrated in experimental models of endotoxin-induced fever (intravenous administration) [12] and kaolin-induced fever, pain and inflammation (s.c. administration) [10]. The superiority of meloxicam injection in cats undergoing ovariohysterectomy was also reported versus placebo and buprenorphine [13,14]. In addition, equivalent efficacy of meloxicam has been reported in cats compared with butorphanol for onychectomy [15], and compared with carprofen, ketoprofen and tolfenamic acid [16], carprofen [17] and tolfenamic acid [13] for ovariohysterectomy. Therefore, there is sufficient evidence published for the efficacy of meloxicam to control pain and inflammation in cats undergoing orthopaedic surgery to justify its use as a positive control for this study. However, in none of the mentioned published studies were the same scoring schemes used as in our study. In optimally designed non-inferiority studies, the methods and outcome measures should be similar to those used in the original studies of the active control [18].

Although the primary objective of the study was the non-inferiority comparison of robenacoxib injection versus meloxicam injection, the effects of follow-up treatment with robenacoxib tablets for approximately 9 days were assessed as a secondary objective. During the following examinations by the clinician at V5 (28 hours post-surgery) and VF, non-inferior efficacy of robenacoxib versus the control group was demonstrated for pain on palpation and overall pain control. Non-inferiority was also shown for inflammation intensity which was assessed at VF. Comparison of robenacoxib tablets versus placebo tablets from Day 1 to VF showed low scores on all days with no significant differences between groups. This result may reflect the possibility that a single pre-surgical administration of robenacoxib or meloxicam was sufficient to control post-operative pain and inflammation in most cats. However, it is also recognised that the methods for assessment of efficacy after Day 1 were not optimal; the veterinarians made only assessments at V5 and VF, and the owner evaluations assessed animal demeanour and general well-being (level of activity, behaviour, appetite, and interaction) and did not monitor anti-hyperalgesic actions. The benefit of administration of robenacoxib tablets for 2 days after surgery in cats was demonstrated statistically versus placebo in another study [7].

Anaesthesia and surgery are both potential stressors. Therefore plasma cortisol, a stressor glucocorticoid biomarker, was measured at four pre-determined times to compare the effects of the two NSAIDs. In both the groups, mean values remained relatively constant at the first three sampling times (T0, T1 and V5) and then increased moderately but significantly at VF. Differences between groups were not statistically significant. Lower plasma cortisol concentrations post-surgery were reported previously in cats receiving meloxicam compared with butorphanol [15], vedaprofen compared with placebo [19] and fentanyl compared to control [20]. However, the relevance of plasma cortisol is not clear, as no differences in plasma cortisol concentrations between surgery and control cats were reported in another study [21].

There were no significant differences in the frequency of reported adverse events in the two groups (*P* = 0.070). Several clinical chemistry and haematology variables increased compared with baseline in both the groups, and these changes were attributed to the anaesthetic protocols and surgical procedures rather than the NSAIDs. The only significant differences between the two groups were higher values for plasma creatinine and urea in the meloxicam group. These results may be type I errors, as the frequency is consistent with the multiple analyses (17 variables at two time points) and an alpha value of 5%.

The rationale for the development of highly COX-2 selective NSAIDs, such as robenacoxib, is that they should offer the same efficacy but better safety than older less selective NSAIDs, such as meloxicam [3]. The finding of no significant differences in tolerability between robenacoxib and meloxicam + placebo in this study is not surprising however, as the study was underpowered to detect differences in safety parameters, with only 147 cats and a relatively short treatment duration (maximum 12 days). In addition, we did not include specific safety investigations, for example gastroscopy, which might have revealed differences in tolerability.

The major limitations of the study are discussed here. First, the scoring schemes used have not been validated in cats. Second, the frequency of assessments was relatively sparse, especially the follow-up from Day 2 onwards, and consisted of daily assessments of the cat’s demeanour and general well-being by the owner. Third, with the exception of the primary end point (the global investigator score), statistical comparisons of efficacy data had to rely on non-parametric statistics because of lack of normal distribution of the data. Reliable non-inferiority analyses could only be performed using RMANCOVA for the primary end point. Fourth, the limitations of non-inferiority studies using positive controls are well known [18]. In our study the proven non-inferiority of robenacoxib versus meloxixam could be due to the fact that both NSAIDs were similarly effective or ineffective, or alternatively that the study lacked sensitivity. It was judged that the use of a placebo pre-surgery would have been unethical, as a number of NSAIDs are registered for pre-operative use in cats in the EU and are widely used. In addition, as noted previously, the efficacy of both meloxicam [10,12] and robenacoxib [3,7] in cats versus placebo has been shown previously A feature of our study was the choice of a non-inferiority threshold (δ) value of 0.25. The δ value should reflect the largest margin that is clinically acceptable, but to date no specific guidelines on δ values for veterinary NSAIDs have been published. In fact, the results show that non-inferior efficacy of robenacoxib to the positive control would also have been achieved if we had defined δ = 0.16 for the primary end point, that is with a maximum of 16% difference in the global investigator score. Furthermore, robenacoxib had numerical superiority to meloxicam for the primary end point (relative efficacy 1.029), which supports the conclusion of non-inferior efficacy.

## Conclusions

Single s.c. injection of robenacoxib (2 mg/kg) before surgery had good tolerability and non-inferior efficacy compared with meloxicam (0.3 mg/kg) for the control of pain and inflammation in cats undergoing orthopaedic surgery. Follow-up treatment with oral robenacoxib tablets for approximately 9 days was well tolerated, but there were no differences in the efficacy scores after Day 1 compared with the group receiving meloxicam s.c. followed by placebo control. This might be the result of single pre-surgical administration of robenacoxib leading to adequate control of pain and inflammation in most cats. However, it is also recognised that the methods for assessment of efficacy after Day 1 were not optimal.

## Endnotes

^a^Onsior® solution for injection 2%, Novartis Animal Health Inc, Basel, Switzerland.

^b^Onsior® non-divisible tablets containing 6 mg of robenacoxib, Novartis Animal Health Inc, Basel, Switzerland.

^c^Metacam® 5 mg/mL injectable, Boehringer Ingelheim Inc, Ingelheim, Germany.

^d^Placebo tablets manufactured by Novartis Animal Health Inc, Basel, Switzerland with identical appearance to the Onsior® tablets

## Abbreviations

ALT: Alanine aminotransferase
AP: Alkaline phosphatase
AST: Aspartate aminotransferase
CI: Confidence interval
CK: Creatine kinase
COX-2: Cyclo-oxygenase-2
C_max_: Maximal concentration
EU: European Union
MRT: Mean residence time
NRS: Numerical rating scale
NSAID: Non-steroidal anti-inflammatory drug
RMANCOVA: Repeated measures analysis of covariance
s.c: Subcutaneous
T: Time
T_max_: Time of maximal concentration
UK: United Kingdom
US: United States
V: Visit
VF: Final visit
